# Proteomic characterization and comparison of the infective and adult life stage secretomes from *Necator americanus* and *Ancylostoma ceylanicum*

**DOI:** 10.1371/journal.pntd.0012780

**Published:** 2025-01-20

**Authors:** Yide Wong, Bruce A. Rosa, Luke Becker, Mali Camberis, Graham LeGros, Bin Zhan, Maria Elena Bottazzi, Ricardo T. Fujiwara, Edita Ritmejeryte, Thewarach Laha, Sujittra Chaiyadet, Piyanan Taweethavonsawat, Paul J. Brindley, Bethany K. Bracken, Paul R. Giacomin, Makedonka Mitreva, Alex Loukas

**Affiliations:** 1 Australian Institute of Tropical Health and Medicine, James Cook University, Cairns, Australia; 2 Centre for Tropical Bioinformatics and Molecular Biology, James Cook University, Cairns, Australia; 3 Department of Medicine, Washington University School of Medicine in St. Louis, St. Louis, Missouri, United States of America; 4 Malaghan Institute of Medical Research, Wellington, New Zealand; 5 National School of Tropical Medicine, Baylor College of Medicine, Houston, Texas, United States of America; 6 Texas Children’s Hospital Center for Vaccine Development, Department of Pediatrics, Baylor College of Medicine, Houston, Texas, United States of America; 7 Department of Parasitology, Institute of Biological Sciences, Universidade Federal de Minas Gerais, Belo Horizonte, Brazil; 8 Department of Parasitology, Faculty of Medicine, Khon Kaen University, Khon Kaen, Thailand; 9 Department of Tropical Medicine, Faculty of Medicine, Khon Kaen University, Khon Kaen, Thailand; 10 Department of Pathology, Faculty of Veterinary Science, Chulalongkorn University, Bangkok, Thailand; 11 Department of Microbiology, Immunology & Tropical Medicine, The George Washington University School of Medicine and Health Sciences, Washington, D.C., United States of America; 12 Charles River Analytics, Cambridge, Massachusetts, United States of America; 13 Macrobiome Therapeutics Pty Ltd, Cairns, Australia; Instituto Oswaldo Cruz, BRAZIL

## Abstract

More than 470 million people globally are infected with the hookworms *Ancylostoma ceylanicum* and *Necator americanus*, resulting in an annual loss of 2.1 to 4 million disability-adjusted-life-years. Current infection management approaches are limited by modest drug efficacy, the costs associated with frequent mass drug administration campaigns, and the risk of reinfection and burgeoning drug resistance. Subunit vaccines based on proteins excreted and secreted (ES) by hookworms that reduce worm numbers and associated disease burden are a promising management strategy to overcome these limitations. However, studies on the ES proteomes of hookworms have mainly described proteins from the adult life stage which may preclude the opportunity to target the infective larva. Here, we employed high resolution mass spectrometry to identify 103 and 57 ES proteins from the infective third larvae stage (L3) as well as 106 and 512 ES proteins from the adult *N*. *americanus* and *A*. *ceylanicum* respectively. Comparisons between these developmental stages identified 91 and 41 proteins uniquely expressed in the L3 ES products of *N*. *americanus* and *A*. *ceylanicum*, respectively. We characterized these proteins based on functional annotation, KEGG pathway analysis, InterProScan signature and gene ontology. We also performed reciprocal BLAST analysis to identify orthologs across species for both the L3 and adult stages and identified five orthologous proteins in both life stages and 15 proteins that could be detected only in the L3 stage of both species. Last, we performed a three-way reciprocal BLAST on the L3 proteomes from both hookworm species together with a previously reported L3 proteome from the rodent hookworm *Nippostrongylus brasiliensis*, and identified eight L3 proteins that could be readily deployed for testing using well established rodent models. This novel characterization of L3 proteins and taxonomic conservation across hookworm species provides a raft of potential candidates for vaccine discovery for prevention of hookworm infection and disease.

## Author summary

Human hookworm infections constitute a significant global health burden, and a vaccine is urgently needed to overcome limitations in current management strategies. No current proteomic study on the secretome of hookworm infective larvae exists and this stage represents a distinct and crucial window where the parasite first encounters the immune system during infection. Identifying excretory/secretory proteins associated with this stage of the lifecycle uncovers novel targets for vaccines and diagnostics. This study comprehensively characterizes and compares excretory/secretory proteins from *Ancylostoma ceylanicum* and *Necator americanus* adult hookworms and infective larvae using mass spectrometry. We found that *A*. *ceylanicum* and *N*. *americanus* infective larvae predominantly express excretory/secretory proteins that are unique to that developmental stage (i.e. not detected in the adult stage secretome) and are likely tailored to the skin and lung environments through which the larvae traverse. This proteome offers a new set of vaccine and diagnostic targets unique to infective hookworm larvae. We also identify several conserved and diversified proteins across both the adult and infective larvae stage of either hookworm. Conserved proteins could represent proteins essential to common functions and may be ideal candidates for a pan-hookworm vaccine.

## 1 Introduction

The human hookworms *Ancylostoma ceylanicum* and *Necator americanus* are the most prevalent species among individuals harboring patent hookworm infections. Recent systematic review of 52 studies reported a global pooled proportion of 79 and 11% respectively [1]. The adult worms are long lived in the human intestines, where they feed on blood and sexually reproduce. Female hookworms release thousands of eggs per day into the intestinal lumen that exit to the environment with the fecal stream. Eggs are deposited into the soil together with host feces, where they hatch, develop and molt twice, maturing into the infective third larvae stage (L3) [2,3]. Skin contact with contaminated soil allows *N*. *americanus* and *A*. *ceylanicum* L3 to enter the host via dermal penetration and migrate to the lung capillaries via the heart. L3 break out of the pulmonary capillaries and creep up the trachea, whereupon they are coughed up, swallowed and make their way through the stomach to the small intestine where they develop into sexually mature male and female adults to continue the life cycle [2,3]. In addition to skin infection, *A*. *ceylanicum* is also transmissible via oral ingestion where L3 are swallowed and migrate directly to the intestines from the stomach [4].

Hookworm infections are a considerable health burden globally. It was estimated that human hookworm disease affected more than 470 million people in 2013 [5] with the loss of 2.1 to 4 million disability-adjusted-life-years and more than $2.4 billion in productivity in 2016 [6]. Intestinal blood loss from hookworm feeding is rarely fatal but can lead to anemia, hypoalbuminemia, and malnutrition, especially in cases of heavy infection and during pregnancy [6–8]. Indeed, hookworm infection was the second most prevalent cause of anemia for both males and females globally in 2010 [9]. Knock on effects from hookworm pathology causes negative effects on physical and cognitive development in children, reduced work capacity and income losses in adults as well as increased risk of pregnancy complications [6,7].

While global efforts to control hookworm infections have been partially successful in reducing prevalence and morbidity, low and middle-income countries continue to face mixed results due to limitations with existing control strategies [5,7,10,11]. For example, targeted deworming of children alone does not reduce the risk of reinfection from the greater community as adults had a greater frequency and intensity of infection [10]. Furthermore, drug regimens targeting soil transmitted helminths (STH) in general such as single dose albendazole or mebendazole may not be effective in curing hookworm infections [7]. Combined with the cost and complications of infrastructure improvements associated with the multifactorial water, sanitation and hygiene (WASH) control programs and the looming threat of hookworm drug resistance [7], there is an urgent need for a human hookworm vaccine to reduce this global health burden.

Both the adult and L3 stages of hookworms excrete and secrete proteins that are essential to parasite infection and survival within the host. Several of these proteins have been investigated as potential human or companion animal vaccines and are currently progressing through various stages of clinical testing [11]. For example, *Na*-GST-1, a glutathione S-transferase in the excretory/secretory proteins (ESP) of adult *N*. *americanus* was found to reduce worm burden in vaccinated hamsters [12]. In phase I clinical trials, *Na*-GST-1 was shown to be well tolerated and effective in generating an IgG response as an adjuvanted vaccine antigen or when co-administered with *Na*-APR-1, an aspartic protease that digests hemoglobin in the hookworm gut [13,14].

To our knowledge, no published reports have investigated and profiled the ES products from the L3 stage of any human hookworm. As the L3 stage also encounters the immune system during its migration from the skin to the lungs, there may be an additional opportunity for a vaccine to target this life stage before it can mature and establish further in the gastrointestinal tract. This study aims to identify, characterize, and compare proteins from the secretomes of the L3 and adult life stages of *N*. *americanus* and *A*. *ceylanicum*. To facilitate follow up studies, we also compare identified hookworm ESPs to a well-studied rodent hookworm, *Nippostrongylus brasiliensis*. The profiles of both conserved and unique secretome proteins from either life stage may provide new insights into hookworm biology and reveal potential vaccine and serodiagnostic candidates.

## 2 Materials and methods

### 2.1 Ethics statement

Ethics approval for infecting Syrian hamster with *N*. *americanus* was obtained from the Animal ethics Committee (CEUA) of the Federal University of Minas Gerais (Protocol# 51/2013) according to the Brazilian Guidelines on Animal Work. Ethics approval for infecting Syrian hamsters with *A*. *ceylanicum* was obtained from the Institutional Animal Care and Use Committee (IACUC) of the Baylor College of Medicine (protocol HA/HC658). Experimental human hookworm infections and egg collection for both *N*. *americanus* and *A*. *ceylanicum* were conducted with written informed consent from all participants according to the National Health and Medical Research Council (NHMRC) National Statement on Ethical Conduct in Human Research under the James Cook University Human Research Ethics Committee (JCU HREC) approval H8339. During the experimental infection study, participants’ health status were monitored via weekly self-assessed symptom scoring and regular lab visits to provide blood samples for pathology tests. Participants were advised on risks of hookworm release into the environment and were advised to maintain sanitation practices. Upon completion of the study, participants were offered the choice to keep their worm infections to maintain the lifecycle for provision of research material or to deworm with over-the-counter deworming medication.

### 2.2 ES products of adult hookworms

Adult *N americanus* ES products were isolated and purified as described [15]. Briefly, *N*. *americanus* adults were recovered at necropsy from Syrian hamsters, washed in PBS and maintained at 37°C, 5% CO_2_ in RPMI 1640 supplemented with 100 μg/ml streptomycin, 100U/ml penicillin and 0.25 μg/ml amphotericin B. Culture supernatant was harvested the next day, centrifuged at 1,500 x *g* for 3 minutes and the supernatant representing the ES products aliquoted and stored at -80°C. Adult *A*. *ceylanicum* were recovered from hamsters at day 40 after infection with 150 *A*. *ceylanicum* L3. Adult worms were similarly maintained (above) in RPMI 1640 supplemented with 100 U/ml ampicillin, 100 μg/ml streptomycin, culture supernatant harvested 24 hours later, clarified by centrifugation at 1,500 x *g* for 10 minutes, passed through a 0.45 μm filter, concentrated, buffer exchanged to PBS pH 7.4 in a Centricon-3 microconcentrator (Amicon, USA), and stored at -80°C.

### 2.3 L3 ES products

Fecal samples were obtained from one healthy volunteer each previously experimentally inoculated with either 10 *A*. *ceylanicum* or *N*. *americanus* L3 larvae via the skin on two separate occasions, approximately 80–90 days between inoculations. The fecal material containing either *A*. *ceylanicum* or *N*. *americanus* eggs were cultured with activated charcoal on 15cm petri dishes at 25°C in a humid chamber for seven days. L3 that migrated to the edge of the dish were harvested and transferred to a 50 ml tube. The larvae were then washed five times in PBS supplemented with 500 μg/ml streptomycin, 500 U/ml penicillin and 1.25 μg/ml amphotericin B (5X Gibco Antibiotic-Antimycotic, Thermo Fisher Scientific, USA), followed by three washes in 200 μg/ml streptomycin, 200 U/ml penicillin and 0.5 μg/ml amphotericin B (2X Gibco Antibiotic-Antimycotic). The larvae were then cultured at 37°C in a tissue culture flask with RPMI 1640 supplemented with 200 μg/ml streptomycin, 200U/ml penicillin and 0.5 μg/ml amphotericin B (2X Gibco Antibiotic-Antimycotic), 2 X L-glutamine and 1% glucose at a concentration of 5000 larvae per ml. Culture supernatant was harvested 48 hours later, centrifuged at 500 x *g*, 2000 x *g* and 4000 x *g* for 30 minutes at 4°C before being stored at -80°C for later use.

### 2.4 S-trap proteomics

Culture supernatants containing ES products were pooled and concentrated and buffer exchanged in 3 kDa Amicon Ultra-15 centrifugal filter with at least four volumes of 50 mM tetraethylammonium bromide (TEAB) at 4°C and 4,000 x *g*. In total, 105 μg of *N*. *americanus* adult ES, 41 μg of *N*. *americanus* L3 ES, 130 μg of *A*. *ceylanicum* adult ES, and 55 μg of *A*. *ceylanicum* L3 ES products split across two replicates were prepared. Samples were lyophilized and solubilized in 50 mM TEAB 5% (w/v) SDS and processed in S-trap micro columns (Protifi, USA) according to the manufacturer’s instructions. In short, solubilized proteins were reduced with 20 mM tris(2-carboxyethyl)phosphine hydrochloride for 15 minutes at 55°C and alkylated with 40 mM iodoacetamide in the dark for 10 minutes at room temperature followed by acidification to a final concentration of around 2.5% (v/v) phosphoric acid and incubation with 100 mM TEAB in 90% (v/v) methanol (binding/wash buffer) to facilitate binding to the S-trap column. Proteins were washed on the S-trap three times with binding/wash buffer and incubated with Solu-trypsin (Sigma, USA) at a 1:10 trypsin to protein ratio for 18 hours. After incubation, peptides were eluted sequentially with 50 mM TEAB, then 0.2% (v/v) formic acid followed by 50% (v/v) acetonitrile and lyophilized for further processing.

### 2.5 Solid-phase sample clean-up

Solid-phase stage tips were prepared by stacking Empore Octadecyl C18 (Supelco/Sigma-Aldrich, USA) disc punches in a 200 μl pipette tip. All centrifugation steps were performed at 1,000 x *g* for at least 1 minute. Stage tips were supported with an adaptor over microtubes and C18 disc punches were centrifuged with 100 μl of methanol followed by 100 μl of 0.2% (v/v) trifluoroacetic acid (TFA) in water. Tryptic peptides eluted from the S-traps were resuspended in 0.2% (v/v) TFA and loaded onto the stage tip over two rounds of centrifugation. Peptides bound to the C18 discs were washed three times with 200 μl of 0.2% (v/v) TFA before a final elution step with 100 μl of 60% acetonitrile 0.2% formic acid (v/v) in water. Eluted peptides were dried, lyophilized, resuspended in 0.1% (v/v) formic acid in water (Solvent A), and submitted for mass spectrometry analysis.

### 2.6 Data acquisition

All samples were analyzed in duplicate with a NanoLC 415 (Eksigent, USA) liquid chromatography system running on Solvent A and 0.1% (v/v) formic acid in 100% HPLC grade acetonitrile (Solvent B) coupled to a TripleTOF 6600 (AB Sciex, USA) mass spectrometer running the Analyst TF 1.8.1 software (Sciex, USA). Digested peptides from all replicates were first loaded on a Proteocol C18 G 3μm 200Å 10 x 0.3 mm trap column (Trajan, Australia) under 10 μl/minute of Solvent A for 5 minutes and separated on a Proteocol C18 G203 3 μm 200Å 250 × 0.3 mm column (Trajan, Australia) with linear gradient of 3–40% solvent B over 90 minutes and at microflow rates of 5 μl/minute. Mass spectrometer parameters were: curtain gas = 35, ion source gas 1 = 25, ion source gas 2 = 30, ionspray voltage floating = 5000, and turboheater temperature = 300°C. Ions were collected and analyzed with 250 ms TOF MS, a collision energy of 5 and a declustering potential of 80 followed by 31 experiments of 50 ms product ion data dependent acquisition scans on product ions with a charge state between 2–5, a collision energy spread of 5, and a declustering potential of 80. The mass windows were 400–1250 Da and 100–1500 Da for the TOF MS and product ion scans, respectively.

### 2.7 Database search and protein identification

Mass spectrometry data files for each replicate were pooled by species and life stage and searched against the respective protein databases reported for *A*. *ceylanicum* [16] and *N*. *americanus* [15] appended with the respective host proteome database from UniProt *Mesocricetus auratus* (hamster) proteome UP000189706 retrieved 24^th^ May 2023 for adult life stages and *Homo sapiens* proteome UP000005640 retrieved 23^rd^ May 2023 for L3 life stages and 381 sequences from the universal protein contaminant library [17] using the Fragpipe V20.0 software [18, 19]. The indexed retention time peptide (Biognosys, Switzerland) sequence was also added to the database as it was added before the acquisition step. Combined databases were appended with an equal number of Fragpipe generated decoy proteins. Searches were performed with the following parameters: precursor mass tolerance of 20 ppm, fragment mass tolerance of 20 ppm, isotype error of 0/1/2, with mass calibration and parameter optimization enabled. Enzyme parameters were set to strict trypsin with two missed cleavages allowed and a peptide length of 7–50 amino acids. Variable modifications of Met oxidation (max of three) and protein N-terminal acetylation with up to a total of three modifications per peptide were allowed. All fixed modifications were enabled. MSBooster was enabled to predict RT and Spectra. PSM validation and Percolator was enabled to with a minimum probability of 0.5. ProteinProphet was set to a maxppmdiff of 2,000,000. The false discovery rate filter was set to 0.01. Search results were further filtered to exclude any proteins from contaminants, the host proteome, and proteins with less than two unique peptides detected.

### 2.8 Protein functionality annotation database construction and functional enrichment testing

All proteins in the current *N*. *americanus* protein set [15] were assigned functional annotations using results from InterProScan v5.42 [20] to identify gene ontology [21] classifications and InterPro functional domains [22], GhostKOALA v2.2 [23] for KEGG [24] annotations, and PANNZER [25] and Sma3s [26] for additional gene naming. Proteins predicted to be secreted were annotated using SignalP v5.0 [27] to identify signal peptides and transmembrane domains. Proteins were characterized as putatively secreted if they contained a signal peptide and contained fewer than two transmembrane domains. The same information was retrieved from a database provided in the previously published *A*. *ceylanicum* annotation [16]. For both species, significant functional enrichment for GO terms was performed using GOSTATS v2.50 [28] and for InterPro domains and KEGG pathways using WebGestalt v2019 [29] (FDR-adjusted *P* ≤ 0.05, minimum two proteins detected, against a background of all annotated proteins for all enrichment testing). For both hookworm species, a comprehensive list of cysteine-rich secretory / SCP/TAPS / CAP domain proteins were identified by searching for associated InterPro domains (IPR035940, IPR001283, IPR014044, IPR035940, IPR035109, IPR002413, and IPR018244).

Proteins with shared orthologs among nematode species were quantified using BLAST [30] (NCBI blastp v2.7.1+, default settings) to identify sequence similarity hits (E ≤ 10^−5^) between each pairwise combination of *A*. *ceylanicum* [16], *N*. *americanus* [15], and *N*. *brasiliensis* [31]. "Reciprocal best BLAST hits" were identified when two proteins from different species were each other’s best match across the proteins. That is, Protein A from species one is best matched to Protein B from species two, and protein B from species two is best matched to protein A from species one. The reciprocal best hit criterion is used to reduce false positives and increase confidence in the assignment of orthologous relationships, and to ensure that results are 1:1 comparable across species. These "reciprocal orthologs" were used to directly compare orthologs across species. In this study, orthologs shared between *A*. *ceylanicum*, *N*. *americanus*, and *N*. *brasiliensis* are referred to as "three-species orthologs". Orthologs of *Schistosoma mansoni* proteins (PRJEA36577.WBPS16 [32]) were also identified for each *A*. *ceylanicum* protein using the same BLAST approach.

Previously-published raw RNA-seq reads for *A*. *ceylanicum* (19 samples across the life cycle stages L3, L4, adult female and adult male [33]) and *N*. *americanus* (two L3 exsheathed and two mixed adult replicates [34]) were downloaded. Trimmomatic v0.36 [35] was used to trim adapters and the STAR aligner [36] (v2.7.5b; 2-pass mode, basic) was used to map RNA-seq reads to the current respective assemblies downloaded from WormBase Parasite [32]. Read counts for every gene in every sample were annotated using the current *A*. *ceylanicum* [16] and *N*. *americanus* [15] gene annotations using featureCounts [37]. For every protein, complete functional annotations, peptide and spectral counts per proteomics sample, BLAST results against other species and normalized gene expression values (FPKM) for all RNA-seq samples are provided for *N*. *americanus* (**S1 Table**) and for *A*. *ceylanicum* (**S2 Table**).

## 3 Results

### 3.1 Proteomic results overview and functional annotation

**Fig 1:**
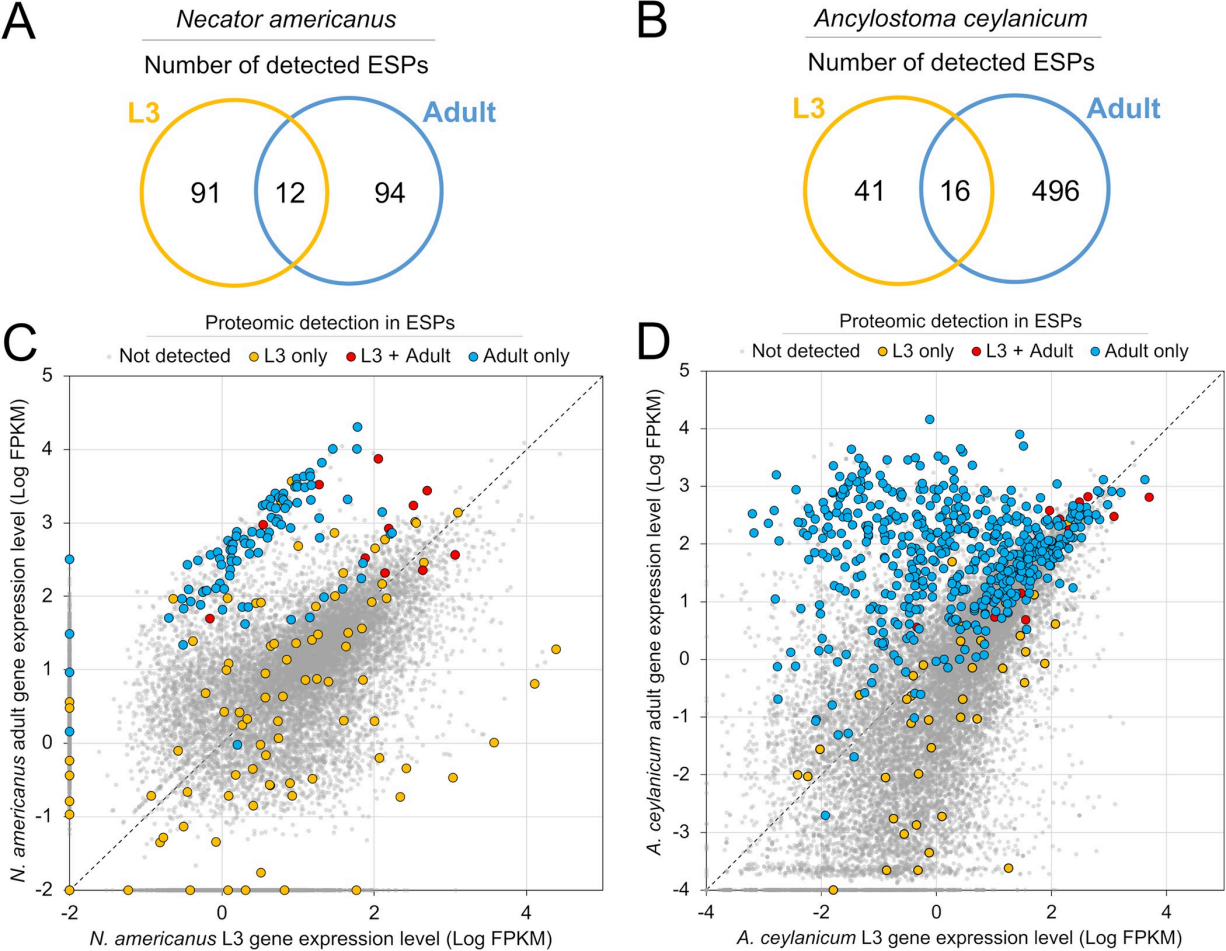
The number of detected ESPs and their relative gene expression levels in L3 and adult stages for both *N*. *americanus* and *A*. *ceylanicum*. Venn diagrams showing the counts of proteins in each stage are shown for (**A**) *N*. *americanus* and (**B**) *A*. *ceylanicum*. The relative gene expression levels of detected proteins and all other genes (based on published datasets) are shown for (**C**) *N*. *americanus* and (**D**) *A*. *ceylanicum*.

Proteomics samples were collected and quantified for samples from ES products from *N*. *americanus* L3 (1,594 spectra identifying 580 peptides in 103 proteins), *N*. *americanus* adult (1,371 spectra identifying 690 peptides in 106 proteins), *A*. *ceylanicum* L3 (936 spectra identifying 290 peptides in 57 proteins), and *A*. *ceylanicum* adults (13,451 spectra identifying 3,441 peptides in 512 proteins). Relative abundance values for each protein are represented by the normalized spectral abundance factor (NSAF), representing total spectral counts normalized to protein length and total spectral intensity in each sample. Among *N*. *americanus* life stages, 91 proteins were L3-specific and 94 were adult-specific ESP, while 12 ESPs were shared between the two stages (**Fig 1A**). For *A*. *ceylanicum*, 41 proteins were L3-specifc compared to 496 adult-specific ESP, while 16 ESPs were shared between these two life cycle stages (**Fig 1B**). Identified proteins were superimposed over a distribution plot of previously published RNA-seq gene expression levels to compare both life stages for *N*. *americanus* [34] (**Fig 1C**) and *A*. *ceylanicum* [33] (**Fig 1D**). In both species, proteins detected only in the adult secretome (blue) tended to have higher gene expression in the adult stage while most proteins detected only in the L3 stage (yellow) had corresponding higher gene expression in the L3 stage. Most proteins detected in both life stages appeared to have overall a relatively high gene expression compared to the other life stage-specific proteins (**Fig 1C** and **1D**, Red).

To facilitate inferred functional characterization and enrichment analysis for detected protein sets of interest, databases were constructed to functionally annotate the current *N*. *americanus* [15] proteome, including InterPro functional domains [22], KEGG enzymes/pathways [24], gene ontology (GO) classifications [21], PANNZER [25], and Sma3s [26] gene naming, and signal peptide annotations (with SignalP [27]). The same data were retrieved from the previously published study of the *A. ceylanicum* ES proteome [16]. Additionally, proteins with shared orthologs among nematode species were analyzed using BLAST [30] to identify reciprocal best BLAST hits among *A*. *ceylanicum* [16], *N*. *americanus* [15], and *N*. *brasiliensis* [31] (i.e., two proteins from different species were each other’s best match across all proteins). All findings including the functional annotations, BLAST statistics, and gene expression data for all proteins are provided for *N*. *americanus* in **S1 Table** and for *A*. *ceylanicum* in **S2 Table**.

### 3.2 Functional characterization of L3-specific hookworm ESPs

Of the 91 proteins unique to the ES from *N*. *americanus* L3 (**Fig 1A**), 53 sequences contained signal peptides for secretion (58.2%, *P* < 10^−15^ for enrichment, binomial distribution test). Among the 16 proteins with a relative protein abundance (NSAF) higher than 0.015, sperm-coating protein (SCP), domain-containing proteins, and Kunitz type protease inhibitors accounted for three proteins each. The top five most abundant proteins in order of relative abundance were annotated as a nematode fatty acid and retinoid binding protein (FAR), a hyaluronoglucosaminidase, a CAP domain protein and two SCP domain-containing proteins (**Table 1A**; the full gene list is provided in **S3 Table**). Among these 91 *N*. *americanus* L3-specific ES products, 14 InterPro domains/families were significantly enriched (**Tables 2A** and **S4A**), including InterPro domains associated with metallopeptidases, CUB domains (most exclusively found in secreted proteins [38]), hyaluronidases, and CAP domains (which were also enriched among the adult-specific ESPs). Five KEGG pathways were significantly enriched in the *N*. *americanus* L3 ESP and four were unique compared to the adult ESP. The KEGG pathway for "peptidases and inhibitors" was significantly and highly enriched (FDR-adjusted *P* <0.001) in both the *N*. *americanus* L3 and adult ESP while the pathways for glycosaminoglycan degradation, betalain biosynthesis, bacterial toxins and isoquinoline alkaloid biosynthesis were uniquely enriched in the L3 ES. A total of 30 molecular function and 24 biological process GO terms were found to be significantly enriched in the ESPs unique to *N*. *americanus* L3, including many peptidase terms also enriched in the adult-specific ESPs, and L3-specific terms including catalytic activity and metal ion binding (**Tables 2A and S4A**).

**Table 1:** The top five most abundant ESPs from protein sets defined in Fig 1A and 1B. Complete protein lists with additional functional annotations are provided in S1 and S1 Tables for *N*. *americanus* and *A*. *ceylanicum* respectively. NSAF = Normalized spectral abundance factor, adjusted for protein length and total number of spectra per sample.

Protein set	Gene / protein ID	Functional annotation	Proteomics					
			L3			Adult		
			Peptide count	Spectral count	NSAF	Peptide count	Spectral count	NSAF
A. *N*. *americanus* L3-specific proteins	NAME_09157	Nematode fatty acid retinoid binding (FAR)	12	104	0.077	0	0	0
	NAME_13301	Hyaluronoglucosaminidase	4	38	0.077	0	0	0
	NAME_06207	CAP superfamily	12	74	0.075	0	0	0
	NAME_01050	SCP domain-containing protein	11	67	0.061	0	0	0
	NAME_14044	SCP domain-containing protein	5	39	0.033	0	0	0
B. *A*. *ceylanicum* L3-specific proteins	ACEY_08746–1	Nematode fatty acid retinoid binding (FAR)	10	96	0.143	0	0	0
	ACEY_04331–1	PPN: Papilin	5	31	0.083	0	0	0
	ACEY_00396–1	ASP1: SCP-like protein	5	39	0.052	0	0	0
	ACEY_14500–1	ASP1: SCP-like protein	12	34	0.030	0	0	0
	ACEY_16636–1	Serine protease inhibitor-like	3	10	0.028	0	0	0
C. *N*. *americanus* Adult*-*specific proteins	NAME_05596	CAP superfamily	0	0	0	3	16	0.048
	NAME_07774	ShTK domain protein	0	0	0	2	15	0.041
	NAME_05396	NECAME_07690 (SCP-like protein)	0	0	0	9	30	0.031
	NAME_14329	SCP domain-containing protein	0	0	0	11	26	0.031
	NAME_13379	*Ancylostoma*-associated secreted	0	0	0	5	14	0.030
D. *A*. *ceylanicum* Adult-specific proteins	ACEY_02018–1	*Ancylostoma*-specific	0	0	0	7	561	0.075
	ACEY_00934–1	*Ancylostoma*-specific	0	0	0	6	229	0.035
	ACEY_17268–1	SCP/CAP domain-containing protein	0	0	0	6	124	0.019
	ACEY_07548–1	ASP3: Secreted protein 3	0	0	0	8	147	0.015
	ACEY_02209–1	*Ancylostoma*-specific	0	0	0	5	78	0.014
E. *N*. *americanus* L3 *& Adult* proteins	NAME_05313	Clade V-conserved protein	4	6	0.010	4	6	0.011
	NAME_01518	TTR-1 (Transthyretin-related-1)	3	6	0.009	3	7	0.012
	NAME_10748	Nematode fatty acid retinoid binding (FAR)	2	4	0.004	5	10	0.012
	NAME_00318	Serine proteinase inhibitor SERPIN B	4	6	0.003	9	18	0.011
	NAME_02523	DVA-1 (DVA-1 polyprotein)	11	15	0.002	59	120	0.016
F. *A*. *ceylanicum* L3 & Adult proteins	ACEY_06526–1	TTR-15 (Transthyretin-like protein 15)	4	9	0.019	8	59	0.007
	ACEY_14447–1	Peptidyl-prolyl cis-trans isomerase B	7	22	0.038	7	29	0.003
	ACEY_01468–1	TTR-16 (Transthyretin-like protein 16)	3	10	0.029	3	22	0.003
	ACEY_11165–1	SERPIN: Serine protease inhibitor	11	45	0.045	14	36	0.002
	ACEY_06670–1	Acey_s0052.g2206	4	6	0.017	6	24	0.004

**Table 2:** Significantly enriched InterPro domains, KEGG pathways and Gene Ontology molecular function and biological process terms among L3-specific ESPs of both *N*. *americanus* and *A*. *ceylanicum*.

Protein set	InterPro domain / KEGG pathway / Gene ontology		Total proteins	Number in protein set	FDR-adjusted *P* value
A. 91 proteins detected only in L3 in *N*. *americanus*	IPR024079	Metallopeptidase, catalytic domain superfamily	132	11	4.1×10^−5^
	IPR001506	Peptidase M12A	83	9	4.3×10^−5^
	IPR006026	Peptidase, metallopeptidase	69	8	8.3×10^−5^
	IPR017050	Metallopeptidase, nematode	29	6	8.3×10^−5^
	IPR035914	Spermadhesin, CUB domain superfamily	50	7	1.0×10^−4^
	IPR017853	Glycoside hydrolase superfamily	60	7	3.0×10^−4^
	IPR000859	CUB domain	46	6	8.2×10^−4^
	*IPR035940	CAP superfamily	158	9	2.6×10^−3^
	IPR003645	Follistatin-like, N-terminal	6	3	2.9×10^−3^
	IPR018155	Hyaluronidase	8	3	7.2×10^−3^
	IPR034035	Astacin-like metallopeptidase domain	45	5	8.2×10^−3^
	*IPR014044	CAP domain	122	7	0.015
	IPR007284	Ground-like domain	28	4	0.015
	IPR002919	Trypsin Inhibitor-like, cysteine rich domain	31	4	0.022
	*ko01002	Peptidases and inhibitors	324	11	6.1×10^−4^
	ko00531	Glycosaminoglycan degradation	17	4	6.1×10^−4^
	ko00965	Betalain biosynthesis	12	3	5.1×10^−3^
	ko02042	Bacterial toxins	12	3	5.1×10^−3^
	ko00950	Isoquinoline alkaloid biosynthesis	17	3	0.012
	*GO:0006508	Proteolysis	391	17	1.2×10^−7^
	*GO:0008233	Peptidase activity	367	15	1.3×10^−6^
	*GO:0004222	Metalloendopeptidase activity	135	10	1.3×10^−6^
	GO:0018996	Molting cycle, collagen, and cuticulin-based cuticle	29	6	2.0×10^−6^
	GO:0042303	Molting cycle	29	6	2.0×10^−6^
	*GO:0004175	Endopeptidase activity	239	12	2.6×10^−6^
	*GO:0008237	Metallopeptidase activity	166	10	4.5×10^−6^
	GO:0005975	Carbohydrate metabolic process	143	9	2.7×10^−5^
	GO:0032501	Multicellular organismal process	80	7	4.1×10^−5^
	GO:0003824	Catalytic activity	2633	36	5.3×10^−5^
	GO:0044238	Primary metabolic process	2075	29	2.4×10^−4^
	*GO:0016787	Hydrolase activity	941	19	2.7×10^−4^
	+ 42 additional GO terms				
B. 41 proteins detected only in L3 in *A*. *ceylanicum*	*IPR002413	Venom allergen 5-like	47	4	0.018
	*IPR001134	Netrin domain	24	3	0.025
	IPR018392	LysM domain	4	2	0.025
	IPR036779	LysM domain superfamily	4	2	0.025
	*IPR001820	Protease inhibitor I35 (TIMP)	29	3	0.031
	(no significant KEGG pathway enrichment)				
	GO:0018958	Phenol-containing compound metabolic process	14	2	1.9×10^−3^
	GO:0044550	Secondary metabolite biosynthetic process	14	2	1.9×10^−3^
	*GO:0004867	Serine-type endopeptidase inhibitor activity	130	4	4.5×10^−3^
	GO:0016716	Oxidoreductase activity. . . incorporation of 1 oxygen atom	13	2	4.5×10^−3^
	*GO:0004866	Endopeptidase inhibitor activity	146	4	4.5×10^−3^
	*GO:0061135	Endopeptidase regulator activity	146	4	4.5×10^−3^
	*GO:0030414	Peptidase inhibitor activity	152	4	4.5×10^−3^
	*GO:0061134	Peptidase regulator activity	156	4	4.5×10^−3^
	GO:0016491	Oxidoreductase activity	412	6	4.5×10^−3^
	*GO:0004857	Enzyme inhibitor activity	159	4	4.5×10^−3^
	GO:0005507	Copper ion binding	18	2	4.5×10^−3^
	+ 14 additional GO terms				

*Also significantly enriched among the proteins detected only in the adult stage

In *A*. *ceylanicum*, of the 41 ESPs unique to the *A*. *ceylanicum* L3 secretome (**Fig 1B**), 31 contained signal peptide sequences (75.6%, *P* < 10^−15^ for enrichment). Among the 12 proteins with an NSAF higher than 0.015, three contained Netrin/Tissue inhibitor of metalloproteinases (TIMP) domains while two were SCP-like proteins. Like *N*. *americanus*, the most abundant protein identified in the *A*. *ceylanicum* L3 secretome was also a nematode FAR (**Tables 1 and S3B**), followed by a papilin, two SCP-like proteins, and a serine protease inhibitor-like superfamily member. Five InterPro domains and families were significantly enriched in the *A*. *ceylanicum* L3 ESP (**Tables 2B and S4B**), but only two were unique to the L3 ESP compared to the adult ESP (two LysM associated terms). The Venom allergen 5-like family (part of the SCP/TPX-1/Ag5/PR-1/Sc7, SCP/TAPS family), Protease inhibitor I35 (TIMP) as well as Netrin InterPro terms were enriched in both *A*. *ceylanicum* L3 and adult ESP. A total of 14 molecular function were significantly enriched in the L3-specific ESPs, five of which were L3-specific (three oxidoreductase activity terms, copper ion binding and monooxygenase activity), and nine terms were also enriched among adult ESPs. These terms shared with adult ESPs primarily consisted of peptidase activity terms, similar to *N*. *americanus*.

### 3.3 Functional characterization of adult-specific hookworm ESPs

Of the 94 adult-specific ESPs in *N*. *americanus* (**Fig 1A**), 54 contained signal peptide sequences (57.4%, *P* < 10^−15^ for enrichment, binomial distribution test), representing a similar proportion as the L3-specific ESPs. In this adult secretome, 20 proteins had a NSAF higher than 0.015, 11 of which were annotated as SCP-like proteins or SCP domain-containing proteins (**S3C Table**). The top five most abundant proteins in order of abundance were a CAP superfamily protein, a Shtk domain protein, a SCP-like protein, a SCP domain-containing protein, and an *Ancylostoma*-associated secreted protein-related protein (**Table 1C**).

In *A*. *ceylanicum*, 277 out of 496 adult-specific ESPs contained signal peptide sequences (55.8%, *P* < 10^−15^), representing a smaller proportion compared to the L3-specific ESPs, although the total number of proteins was much higher in adults. Overall NSAF values were lower due to the higher number of proteins, but nine proteins had a NSAF higher than 0.01, with four of these annotated as CAP domain proteins, three as *Ancylostoma*-specific proteins, two annotated as SCP domain-containing proteins, and another two as *Ancylostoma*-associated secreted proteins (ASPs; **S3D Table**). Of note, some overlap was present between annotations, and among the nine most abundant proteins, two SCP domain-containing proteins, and one *Ancylostoma*-associated secreted protein were also annotated as CAP domain proteins. Lastly, the two most abundant identified proteins were *Ancylostoma*-specific proteins, with no other known functional annotations assigned (**Table 1D**).

Enriched InterPro domains among the adult-specific ESPs were similar for the two species (**Tables 3A, 3B**, **S4C and S4D**, and included very strong enrichment for CAP domains (*P* < 10^−15^), peptidases and inhibitors (*P* ≤ 4.1×10^−5^) and peptidase activity (*P* ≤ 2.7×10^−9^).

### 3.4 Functional characterization of ESPs present in both L3 and adult stages

The 12 proteins detected in both the L3 and adult ESPs in *N*. *americanus* (**Fig 1A**) are provided in **S3E Table,** and the 16 proteins detected in both the L3 and adult ESPs in *A*. *ceylanicum* (**Fig 1B**) are provided in **S3F Table**. The ESPs in both species include transthyretin-like proteins, FARs, galectins and serine proteinase inhibitors, while *N*. *americanus* ESPs also contain a DVA polyprotein and an immunogenic protein 3, while *A*. *ceylanicum* ESPs contain an enolase and a ferritin. The top five most abundant ESPs in both stages are shown in **Table 1E** for *N*. *americanus* and **Table 1F** for *A*. *ceylanicum*.

**Table 3:** Significantly enriched InterPro domains, KEGG pathways and Gene Ontology molecular function and biological process terms among adult-specific ESPs of both *N*. *americanus* and *A*. *ceylanicum*.

Protein set	InterPro domain / KEGG pathway / Gene ontology		Total proteins	Number in protein set	FDR-adjusted *P* value
A. 94 proteins detected only in adult in *N*. *americanus*	*IPR035940	CAP superfamily	158	40	<10^−15^
	*IPR014044	CAP domain	122	33	<10^−15^
	IPR034164	Pepsin-like domain	24	7	1.1×10^−6^
	IPR001461	Aspartic peptidase A1 family	25	7	1.1×10^−6^
	IPR001283	Cysteine-rich secretory, allergen V5/Tpx-1	39	8	1.1×10^−6^
	IPR033121	Peptidase family A1 domain	33	7	7.1×10^−6^
	IPR021109	Aspartic peptidase domain superfamily	65	7	7.8×10^−4^
	IPR018244	Allergen V5/Tpx-1-related, conserved site	29	5	1.8×10^−3^
	IPR008632	Nematode fatty acid retinoid binding	9	3	0.015
	IPR001747	Lipid transport protein, N-terminal	3	2	0.049
	IPR015816	Vitellinogen, beta-sheet N-terminal	3	2	0.049
	*ko01002	Peptidases and inhibitors	324	10	4.1×10^−5^
	ko00471	D-Glutamine and D-glutamate metabolism	5	2	0.029
	ko04210	Apoptosis	87	4	0.034
	ko04142	Lysosome	167	5	0.034
	*GO:0008233	Peptidase activity	367	15	2.7×10^−9^
	*GO:0004175	Endopeptidase activity	239	12	1.8×10^−8^
	GO:0070001	Aspartic-type peptidase activity	45	7	1.8×10^−8^
	GO:0004190	Aspartic-type endopeptidase activity	45	7	1.8×10^−8^
	*GO:0006508	Proteolysis	391	13	4.0×10^−7^
	*GO:0061135	Endopeptidase regulator activity	82	6	1.9×10^−5^
	*GO:0004866	Endopeptidase inhibitor activity	82	6	1.9×10^−5^
	*GO:0061134	Peptidase regulator activity	88	6	2.1×10^−5^
	*GO:0030414	Peptidase inhibitor activity	88	6	2.1×10^−5^
	+17 additional GO terms				
B. 496 proteins detected only in adult in *A*. *ceylanicum*	IPR035940	CAP superfamily	464	104	<10^−15^
	IPR014044	CAP domain	393	95	<10^−15^
	IPR001283	Cysteine-rich secretory protein-related	305	72	<10^−15^
	IPR035109	*Ancylostoma*-associated secreted protein related	135	23	8.2×10^−7^
	IPR008993	Tissue inhibitor of metalloproteinases-like (TIMP)	43	12	1.7×10^−5^
	IPR024079	Metallopeptidase, catalytic domain superfamily	187	25	1.7×10^−5^
	IPR001506	Peptidase M12A	122	19	5.7×10^−5^
	IPR025660	Cysteine peptidase, histidine active site	46	11	2.5×10^−4^
	+41 additional IPR domains				
	ko01002	Peptidases and inhibitors	405	45	<10^−15^
	ko04147	Exosome	577	49	1.1×10^−11^
	ko04612	Antigen processing and presentation	72	14	5.7×10^−7^
	ko04210	Apoptosis	107	16	1.5×10^−6^
	ko03110	Chaperones and folding catalysts	205	22	1.5×10^−6^
	ko00536	Glycosaminoglycan binding proteins	125	16	1.1×10^−5^
	+34 additional KEGG pathways				
	*GO:0006508	Proteolysis	572	60	9.0×10^−18^
	GO:0008233	Peptidase activity	531	55	1.1×10^−13^
	GO:0008152	Metabolic process	2958	129	7.2×10^−12^
	GO:1901564	Organonitrogen compound metabolic process	1672	88	6.4×10^−9^
	GO:0071704	Organic substance metabolic process	2846	121	9.2×10^−9^
	GO:0016787	Hydrolase activity	1238	78	1.4×10^−8^
	GO:0004175	Endopeptidase activity	358	36	1.7×10^−8^
	GO:0003824	Catalytic activity	3127	143	7.5×10^−8^
	GO:0008237	Metallopeptidase activity	248	28	8.4×10^−8^
	GO:0004222	Metalloendopeptidase activity	204	25	9.6×10^−8^
	GO:0044281	Small molecule metabolic process	353	32	3.1×10^−7^
	GO:0046872	Metal ion binding	727	50	1.3×10^−6^
	+97 additional GO terms				

*Also significantly enriched among the proteins detected only in the L3 stage

### 3.5 ESPs conserved across hookworm species

Shared orthologs were identified using best reciprocal BLAST hits between each pairwise combination of *A*. *ceylanicum* [16], *N*. *americanus* [15], and *N*. *brasiliensis* [31] proteins. "Reciprocal best BLAST hits" were identified when two proteins from different species were each other’s best match across the proteins.

**Fig 2:**
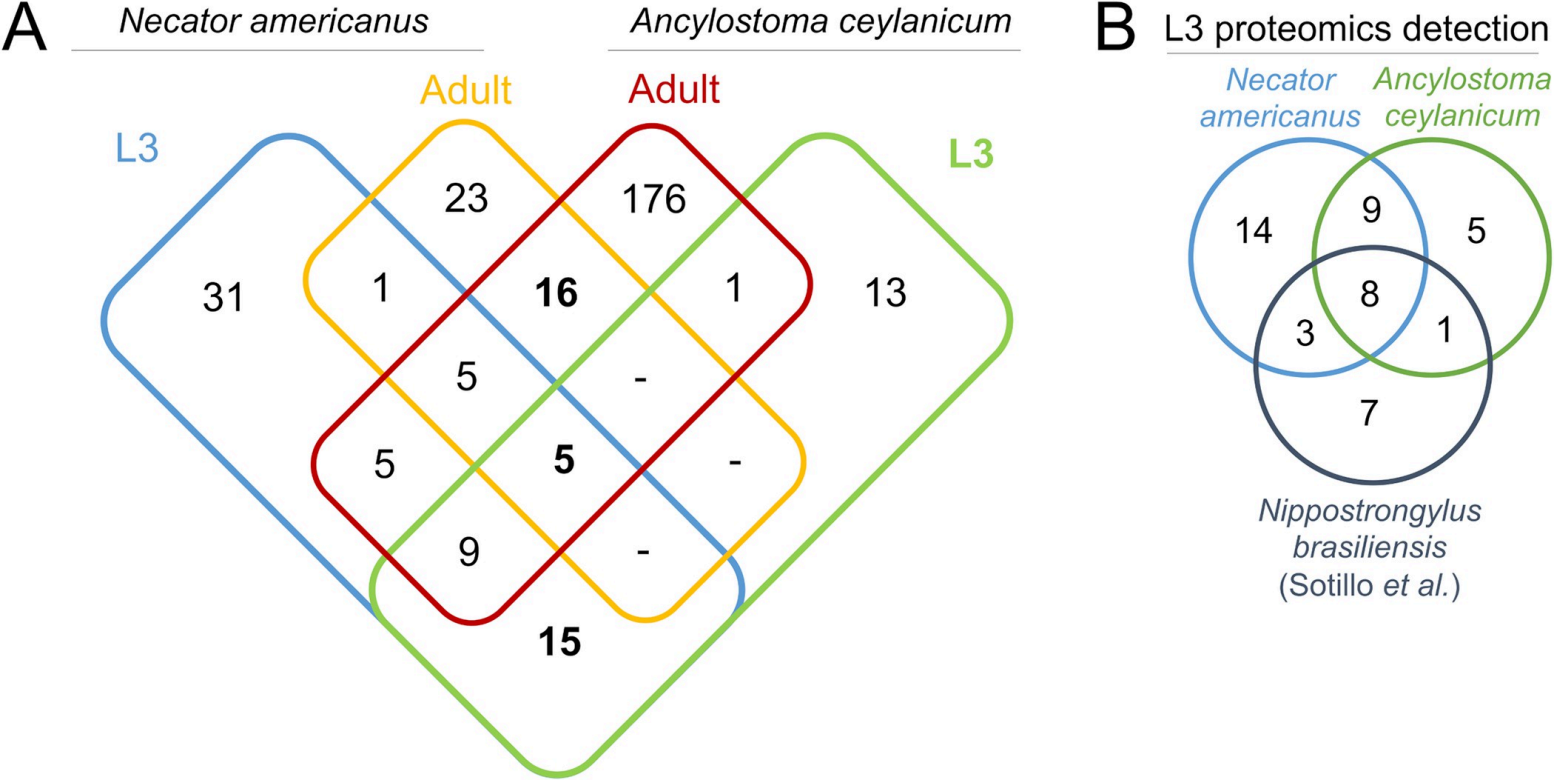
ESP counts in cross-species comparisons, matched by 1:1 reciprocal best BLAST orthologs. (**A**) Overlap of L3 and adult ESPs from *N*. *americanus* and *A*. *ceylanicum* from the current study. (**B**) Overlap of L3 ESPs from *N*. *americanus*, *A*. *ceylanicum*, and *N*. *brasiliensis* (from Sotillo et al. 2014). (**C**) Overlap of adult *N*. *americanus* ESPs (current study and Logan et al. 2020) and *A*. *ceylanicum* (current study and Uzoechi et al. 2023).

Fifteen orthologous proteins were detected only in the L3 secretomes of *N*. *americanus* and *A*. *ceylanicum* but not in the adult stages of these two species (**Fig 2A**, **Tables 4A** and **S3G**). Ten (66.6%) of these proteins contained a signal peptide for secretion, two sequences were annotated as tyrosinases, and another two proteins remained functionally unannotated. The top five most abundant proteins in order of abundance were annotated as papilin, TIMP, zinc metalloproteinase NAS-28, glutathione peroxidase, and a ground-like domain-containing protein. Sixteen orthologous protein pairs were detected only in the adult secretomes of *N*. *americanus* and *A*. *ceylanicum* (**Fig 2A**, **Tables 4B** and **S3I**). Thirteen (81.2%) sequences contained a signal peptide for secretion and two were inferred as neprilysin metalloproteases. Four proteins were inferred as CAP domain proteins and two of these CAP domain proteins were also inferred as SCP-like proteins. The remaining two were inferred as a CRISP1/2/3 protein. The top five most abundant orthologs among *N*. *americanus* and *A*. *ceylanicum* adult secretomes were a LYS-8 protein, FAR-1, a SCP-like protein, a transthyretin-like family protein and a glutamate dehydrogenase. Lastly, only five sequences were identified as orthologs detected in the secretomes of both life stages and hookworm species (**Fig 2A**, **Tables 4C** and **S3H**), and four (80%) sequences contained signal peptides. These proteins were annotated as a transthyretin-related-1 protein, a clade V-conserved protein, a transthyretin-like family protein, galectin, and a FAR.

**Table 4:** Conserved stage-specific ESPs of *N*. *americanus* and *A*. *ceylanicum*.

Gene set	*N*. *americanus* ortholog	Best annotation	*A*. *ceylanicum* ortholog	Proteomic abundance (NSAF)			
				*N*. *americanus*		*A*. *ceylanicum*	
				L3	Adult	L3	Adult
A. L3 and not adult stages	NAME_03234	PPN (Papilin)	ACEY_04331–1	0.0058	0	0.083	0
	NAME_05503	Tissue inhibitor of metalloproteinase (TIMP)	ACEY_18090–1	0.0084	0	0.019	0
	NAME_08728	NAS-28 (Zinc metalloproteinase nas-28)	ACEY_01795–1	0.0095	0	0.012	0
	NAME_02640	GPX1 (Glutathione peroxidase)	ACEY_15667–1	0.0017	0	0.019	0
	NAME_11085	Ground-like domain-containing protein	ACEY_14558–1	0.0075	0	0.0079	0
	NAME_12773	TYR: tyrosinase	ACEY_00264–1	0.0066	0	0.0079	0
	NAME_09579	IFI30, GILT: interferon, gamma-inducible 30	ACEY_02509–1	0.0093	0	0.0049	0
	NAME_08112	RPB1 (DNA-directed RNA polymerase subunit)	ACEY_04109–1	0.0017	0	0.012	0
	NAME_04621	TYR: tyrosinase	ACEY_16687–1	0.0064	0	0.0060	0
	NAME_08546	-	ACEY_04726–1	0.0077	0	0.0047	0
	NAME_02096	DDO-3 (D-aspartate oxidase 3)	ACEY_01668–1	0.0034	0	0.0077	0
	NAME_00148	Peptidase, S9A/B/C family protein	ACEY_04574–1	0.0054	0	0.0014	0
	NAME_06985	-	ACEY_10391–1	0.0023	0	0.0037	0
	NAME_00763	Transmembrane matrix receptor MUP-4	ACEY_05489–1	0.0020	0	3.6×10^−4^	0
	NAME_13229	CBG00717 (Hint module)	ACEY_15810–1	0.0017	0	5.6×10^−4^	0
B. Adult and not L3 stages	NAME_02556	LYS-8 (Protein CBR-LYS-8)	ACEY_07461–1	0	0.020	0	6.6×10^−4^
	NAME_07503	FAR-1 (Fatty-acid and retinol-binding protein 1)	ACEY_03045–1	0	0.013	0	0.0053
	NAME_11454	SCP-like protein (CAP domain)	ACEY_03757–1	0	0.0092	0	0.0061
	NAME_14211	TTL1 (Transthyretin-like family protein)	ACEY_16947–1	0	0.0093	0	0.0050
	NAME_02908	GLDH (Glutamate dehydrogenase)	ACEY_05952–1	0	0.0054	0	0.0062
	NAME_01070	CRISP1/2/3 (CAP domain)	ACEY_02230–1	0	0.0093	0	0.0014
	NAME_12492	TFPI: tissue factor pathway inhibitor	ACEY_07361–1	0	0.0083	0	0.0018
	NAME_04983	SCP-like protein (CAP domain)	ACEY_06324–1	0	0.0049	0	0.0031
	NAME_00885	Neprilysin / MEP1 (Zinc metallopeptidase 1)	ACEY_00118–1	0	0.0057	0	0.0015
	NAME_00886	Neprilysin / MEP1b (Peptidase family M13)	ACEY_00117–1	0	0.0049	0	0.0012
	NAME_13076	CRISP1/2/3 / ASP5 (CAP domain)	ACEY_00798–1	0	0.0038	0	8.3×10^−4^
	NAME_00850	-	ACEY_12608–1	0	0.0037	0	6.8×10^−4^
	NAME_09695	Eukaryotic aspartyl protease	ACEY_00578–1	0	0.0020	0	9.2×10^−4^
	NAME_00535	NPEPPS: puromycin-sensitive aminopeptidase	ACEY_18305–1	0	5.1×10^−4^	0	0.0013
	NAME_09716	VIT (Vitellogenin)	ACEY_14669–1	0	0.0009	0	5.2×10^−4^
	NAME_00332	Cathepsin D / NCPI (Eukaryotic aspartyl protease)	ACEY_13381–1	0	0.0010	0	1.4×10^−4^
C. Adult and L3 stages	NAME_01518	TTR-1 (Transthyretin-related-1)	ACEY_06526–1	0.0089	0.012	0.019	0.0068
	NAME_05313	Clade V-conserved protein	ACEY_06670–1	0.010	0.011	0.017	0.0037
	NAME_02187	CBG05997 (Transthyretin-like family)	ACEY_01468–1	0.0043	0.0029	0.029	0.0035
	NAME_04727	LEC-5 (Galectin)	ACEY_07349–1	0.0087	0.0026	0.015	0.0036
	NAME_10748	CBG06811 (Nematode fatty acid retinoid binding)	ACEY_01256–1	0.0043	0.012	0.0051	0.0022

Identified L3 secretome orthologs from *N*. *americanus* and *A*. *ceylanicum* were compared to the *N*. *brasiliensis* L3 secretome [31] to further identify three-species reciprocal orthologs. Of 29 reciprocal hits identified from the *N*. *americanus* and *A*. *ceylanicum* L3 secretomes (**Fig 2A**), eight were detected in reciprocal orthologs from the *N*. *brasiliensis* ES (**Fig 2B**). Some notable proteins include brorin, extracellular superoxide dismustase as well as NAS-28 and D-aspartate oxidase 3 (DDO-3), both of which were L3-specific reciprocal hits not detected in the *N*. *americanus* and *A*. *ceylanicum* adult secretomes (**Table 5**).

**Table 5:** L3 stage-specific ESP orthologs also detected in *N*. *brasiliensis*.

*A*. *ceylanicum* ortholog	Best annotation	Signal peptide
ACEY_18213–1	SOD: Extracellular superoxide dismutase [Cu-Zn]	Y
ACEY_00713–1	Kunitz/Bovine pancreatic trypsin inhibitor domain protein	Y
ACEY_01795–1	NAS-28: Zinc metalloproteinase nas-28	Y
ACEY_18090–1	Netrin / Tissue inhibitor of metalloproteinase (TIMP)	-
ACEY_00822–1	VWC2: brorin	Y
ACEY_16687–1	Bm3483: ShTK domain protein	Y
ACEY_14447–1	PPIB: Peptidyl-prolyl cis-trans isomerase B	Y
ACEY_01668–1	DDO-3: D-aspartate oxidase 3	Y

### 3.6 Comparison to *Schistosoma* cercariae ESPs, also secreted during skin invasion

The cercaria stage of the flatworm *Schistosoma* also enters the host by invasion through the skin during the cercarial stage [39], so comparisons to ESP proteomes from this stage of *Schistosoma* may give insights into broader helminth-conserved skin invasion mechanisms. There are two such available proteomic studies of *S*. *mansoni* cercariae ESPs [40,41], but these predate the availability of the *S*. *mansoni* genome annotation, and many of the Uniprot IDs identified are long deprecated, so systematic 1:1 comparisons are difficult, but manual searching of the tables from the studies was performed using BLAST orthologs to *S*. *mansoni* proteins (PRJEA36577). No *S*. *mansoni* orthologs were identified among the five proteins detected in ESPs from both stages and both species of hookworms, but five of the nine ESPs detected in L3 in both hookworm species and adult-stage *A*. *ceylanicum* ESPs had significant *S*. *mansoni* orthologs. These included (i) enolase and (ii) aldolase/fructose-bisphosphate aldolase, the two most abundant proteins identified from lipid-induced cercarial secretions and among the most highly released from acetabular glands in Knudsen et al. 2005 [40], and both were detected in cercarial ESPs in Curwen et al. 2006 [41]. The other three proteins include (iii) peptidyl-prolyl cis-trans isomerase (detected in lipid-induced cercarial and acetabular gland secretions [40], and in ESPs in Curwen et al. 2006 [41]), (iv) superoxide dismutase (detected in lipid-induced cercarial secretions [40]) and (v) Smp_089670/ SM21.7 (detected in lipid-induced cercarial and acetabular gland secretions [40]). Finally, three orthologs of *S*. *mansoni* proteins were among the 15 proteins detected only in L3 in both hookworm species: (i) dipeptidyl peptidase / dpf-1, one of 16 proteins detected in cercariae ESPs, (ii) D-aspartate oxidase, and (iii) papilin/spondin, neither of which were detected in the previous studies. Both studies identified elastases as major abundant components of *S*. *mansoni* cercariae ESPs [40, 41], but no orthologs of these or any proteins annotated as elastases were identified in either hookworm species. Together, these results indicate several sets of proteins that appear to be very important for skin penetration, including helminth-conserved proteins (especially enolase and adolase), nematode-specific proteins (transthyretin proteins, FAR-1, galectins, and others) and enolases as very abundant trematode-specific proteins. BLAST results between *A*. *ceylanicum* and *S*. *mansoni* proteins used for this analysis are provided in **S2 Table**.

## 4 Discussion

Given that commercial hookworm vaccines remain unavailable [42], continued research and development in this field is a global public health priority. As noted, efforts to develop a hookworm vaccine have focused on antigens secreted by the adult worms, which lead to development of the lead candidates *Na*-ASP-2 and *Na*-APR-1. However, both so far have limitations [43,44], circumstances which support the merit of continued discovery-focused research programs aimed at the identification of new vaccine targets. To this end, here we have generated and characterized comprehensive proteomic profiles from the secretomes of the L3 and adult life stages of two of the major human hookworms, *N*. *americanus* and *A ceylanicum*. Broadly, we find minimal overlap between proteins identified from the L3 and adult secretomes from both hookworms. Proteins shared between life stages in either species accounted for only 11.6% and 11.3% of the L3 and adult *N*. *americanus* ES proteomes, and 28% and 3.1% of the L3 and adult *A*. *ceylanicum* ES proteomes, respectively (**Fig 2**). This finding is congruent with data from mass-spectrometric analysis in other nematodes that showed proteins shared between the secretomes of infective L3 and adult life stages were only a relatively smaller portion of their respective proteome. Comparison of proteins identified from the *N*. *brasiliensis* L3 and adult secretomes also revealed an overlap between life stages of 25% and 4.9% of the L3 and adult ESPs, respectively [31]. The proteins shared between the infective L3 ESPs and the combined hydro-soluble plus hydro-insoluble female adult ESPs of *Strongyloides stercoralis* consisted of 19.7% and 43.8% of the respective developmental stage secretomes [45].

Perhaps the relatively small number of proteins identified in both developmental stages may be due to the lower number of proteins identified here in the adult secretomes compared to previous studies, which identified more unique proteins from the secretomes of adult *N*. *americanus* [15] and *A*. *ceylanicum* [16]. As those studies used the same protein sequence library for their spectral searches, we were able to compare L3 proteins identified here against the published adult secretomes. Comparison of the *N*. *americanus* ESPs with the 197 adult ESPs identified by Logan et al. 2020 [15] showed that 83.0% of our 106 adult-stage proteins were also previously detected and 89% of our 91 L3-specific ESPs had not been detected in adult-stage ES (**S1 Table**). Comparison of the *A*. *ceylanicum* ESPs from this study with the recently published adult secretome by Uzoechi et al. 2023 [16] showed that 81.6% of our 512 adult-stage ESPs were also previously detected and 92.7% of our 40 L3-specific proteins had not been detected in adult-stage ES (**S2 Table**). It is also possible that the different culture conditions caused the large differences in detected ESP from the two species of adult hookworms in this study. However, the study by Uzoechi et al. 2023 which cultured adult *A*. *ceylanicum* under different conditions also identified many more ESPs relative to both the previous and current report from adult *N*. *americanus* [15,16]. *A*. *ceylanicum* is known to cause a larger volume of blood loss per worm during infection compared *N*. *americanus* [4] and it is possible that its feeding habits may result in the secretion and excretion of a more extensive cocktail of proteins. Overall, these findings confirmed a high level of consistency with the adult ES from previous studies, which buttresses our interpretation that the L3 secretome from *N*. *americanus* and *A*. *ceylanicum* are both markedly dissimilar to the adult secretome. Considering the behavioral and environmental lifestyle differences between tissue invasive, non-feeding larvae and reproductive feeding adults, the findings here are not unexpected.

### Metalloproteinases

Two biological process GO terms associated with the molting cycle were uniquely and highly enriched in the *N*. *americanus* L3 ESPs (**Table 2A**; "molting cycle, collagen and cuticulin-based cuticle", *P* = 2.0×10^−6^ and "molting cycle", *P* = 2.0×10^−6^), with six of 103 proteins assigned these terms. These six proteins were also linked to GO terms associated with zinc ion binding, proteolysis, and metallopeptidase activity. Inferred as either various types of metalloendopeptidases or metalloproteinases such as MTP-1, HCH-1, or NAS-28, all six proteins were also shown by InterProScan analysis to belong to the peptidase family M12; four belonged to the subgroup of astacin-like metallopeptidases, while all but HCH-1 would contain the CUB domain structural motif. Although it did not have an NSAF higher than 0.015, a NAS-28 astacin-like metallopeptidase associated with the molting cycle GO term was identified with the 16^th^ highest relative abundance among *A*. *ceylanicum* L3-specific proteins. Further, this NAS-28 was found to be ortholog between the L3 secretomes from both hookworms in this study and the previously published *N*. *brasiliensis* L3 secretome [31].

Astacin-like metalloproteases containing a CUB domain were shown to be crucial in cleaving cuticle collagen for successful molting in the free-living nematode *Caenorhabditis elegans* [46]. Disrupting the expression of such proteins resulted in body morphology or molting defects, as these proteins appear to be required for cuticle formation and shedding [46–48]. Additionally, rescue with the respective protein orthologs from the parasitic nematodes *Haemonchus contortus* and *Brugia malayi* was able to correct the defective phenotype, suggesting that the purpose of these proteins may be conserved across different free-living and parasitic nematode species [49, 50]. The collagenolytic activity of metalloproteases was also demonstrated to be important for tissue invasion as an astacin-like metalloprotease, *Ac*-MTP-1 from the dog hookworm *Ancylostoma caninum* was shown to degrade connective tissue substrates gelatin, fibronectin and laminin, and exposure to metalloprotease inhibitors significantly reduced the number of *A*. *caninum* L3 that penetrated skin *in vitro* [51]. Furthermore, vaccination of dogs with recombinant *Ac*-MTP-1 followed by infection challenge with *A*. *caninum* resulted in worm burdens and egg counts that were inversely correlated with anti-Ac-MTP-1 IgG2 titers [52].

### Oxidoreductases and Glycoside hydrolases

Several other functional processes were enriched across the L3 secretome profiles. Notably, the molecular function GO term "oxidoreductase activity" was uniquely enriched in the L3 secretomes from both *N*. *americanus* and *A*. *ceylanicum* (**S4A** and **S2B Tables**; *P* = 0.010 and *P* = 4.5×10^−3^, respectively). Abundant proteins associated with this enrichment include D-aspartate oxidase (DDO-3), which catalyzes the transfer of electrons from reductant to oxidant molecules, and can facilitate different biological processes in nematodes or cestodes such as protecting against oxidative damage, facilitating the metabolism of D-amino acids, and neurotransmission [53–56]. DDO-3 was identified among the orthologous proteins of both the *N*. *americanus* and *A*. *ceylanicum* L3 secretomes, and *N*. *brasiliensis* L3 secretome, suggesting that this protein could be crucial to hookworm invasion and survival [31].

Proteins annotated with the glycoside hydrolase (GH) superfamily InterPro domain were uniquely enriched in the *N*. *americanus* L3 secretome (**Table 2A**; *P* = 3.0×10^−4^). GH family enzymes act on glycosidic bonds, and we identified a protein annotated as family 31 glycosyl hydrolase (GH31). GH31 family members are highly expressed in *Ascaris suum* ES products, where they may degrade and metabolize host carbohydrates [57]. Indeed, several proteins identified in the *N*. *americanus* L3 secretome that belonged to this family were also functionally mapped to the GO term of carbohydrate metabolic processes. For example, α-L-fucosidase is critical for biological processes such as embryo development, mobility and longevity in the pine wood nematode *Bursaphelenchus xylophilus* [58], while malate dehydrogenase participates in the citric acid cycle [59].

Hyaluronidases are a subset of the glycoside hydrolase superfamily as they cleave the β1–4 glycosidic linkages in hyaluronic acid [59,60]. Hyaluronidases facilitate skin invasion and enable hookworm larvae passage between keratinocytes through hydrolysis of extracellular matrix hyaluronic acid [61, 62]. We found that the hyaluronidase functional term was notably enriched in the *N*. *americanus* L3 secretome (**Table 2A**; *P* = 7.2×10^−3^). Intriguingly larval homogenates from the canine hookworm *A*. *braziliense* exhibit far greater hyaluronidase activity than *Ancylostoma tubaeforme* and *A*. *caninum* [61]. This disparity might be attributed to the route of infection as *A*. *tubaeforme* and *A*. *caninum* can infect via both the skin and oral routes whereas *A*. *braziliense* is an obligate skin-penetrator [61]. We have not detected proteins with hyaluronidase activity from the *A*. *ceylanicum* L3 secretome, and given that *A*. *ceylanicum* is infective via oral or skin routes, it might secrete less hyaluronidase than *N*. *americanus* [4].

### Cysteine-rich secretory proteins

Numerous sequences inferred as cysteine-rich secretory proteins (CRISP), *Ancylostoma*-secreted proteins (ASP), SCP-like or SCP-domain containing proteins belonging to the CAP superfamily were identified across both hookworm species and life stages in this study. The CAP superfamily is also known as the SCP protein family and it encompasses a diverse group of proteins defined by the presence of cysteine-rich CAP motifs which make up structurally conserved disulfide stabilized CAP (sometimes referred to as SCP or PR-1) domains of 17–21 kDa in size [63]. Proteins of this family are often referred to as SCP/TPX-1/Ag5/PR-1/Sc7 (SCP/TAPS) or ASP proteins [15,16,64]. SCP/TAPS proteins are highly expressed in the ES products of the larval and adult developmental stages of numerous helminths [15, 16, 31, 45, 63, 65–68]. Functional terms associated with the venom allergen 5-like protein (VAL) family were also enriched in the *N*. *americanus* adult secretome and the secretome from both life stages of *A*. *ceylanicum*. A subset of SCP/TAPS, VALs appear to preferentially bind hydrophobic molecules such as sterols or fatty acids [69, 70].

Both VALs and the broader family of SCP/TAPS proteins in helminths associate with activities such as immune modulation [71, 72], host invasion, and transition to parasitism [73–75]. Some ASPs or VALs confer vaccine efficacy [76, 77], although vaccination with *Na*-ASP-1 induced an IgE mediated urticaria in endemic region study volunteers with pre-existing hookworm protein-specific IgE [72, 78]. Despite the significance and enrichment of this protein family, little is known about their molecular mechanisms of action, and research is needed to elucidate its functional contribution to hookworm biology [64].

In this study, we detected 124 of these cysteine-rich secretory proteins in *A*. *ceylanicum* adult, four in *A*. *ceylanicum* L3, 43 in *N*. *americanus* adult and 11 in *N*. *americanus* L3. All of the cysteine-rich secretory proteins identified in each species are identified in **S1** and **S2 Tables**.

### Additional ESPs of interest

The potential development of vaccines based the identification of orthologous proteins from both larval and adult stages presents an opportunity for pan-hookworm protection. The orthologous proteins from the *N*. *americanus* and *A*. *ceylanicum* L3-specific secretome (**Table 4A**) provide a larger pool of novel immunization candidates not detected in the adult secretome, that could target the larvae during its parenteral migration. Indeed, a recent clinical trial with repeated rounds of short-term exposure with *N*. *americanus* L3 followed by anthelmintic treatment induced a protective response that was associated with skin responses, indicating that antibody-mediated killing of L3 in the skin generates meaningful immunity [79]. Furthermore, the three-species L3 orthologs offer a group of proteins that have been down-selected from the profiled L3 secretome that can readily be evaluated in the *N*. *brasiliensis* mouse challenge model due to the presence of orthologs in the *N*. *brasiliensis* secretome. Aside from the previously discussed DDO-3 and NAS-28, a tissue inhibitor of metalloproteases (TIMP/Netrin) and a tyrosinase copper-binding oxidoreductase containing a Shtk domain were identified among the three-species orthologs. Furthermore, TIMP/Netrins were highly represented in the proteins unique to the *A*. *ceylanicum* secretome and another tyrosinase/Shtk domain protein was identified among ESP orthologs from all three species.

As reflected in its name, TIMPs inhibit metalloproteases. Their N-terminal domains share structure with netrin domains, which results in overlap between the TIMP and Netrin functional annotations in this study [80, 81]. However, sequence similarity may not translate to functional activity. AceES-2, previously identified from *A*. *ceylanicum* was shown to be structurally similar to other netrin domain-containing TIMPs yet does not inhibit matrix metalloproteases [82]. Due to their structural properties, the immunomodulatory activity of TIMPs may be due to cytokine binding, and therefore unrelated to the inhibition of metalloprotease catalysis [82–85]. Whereas the precise functions of tyrosinases and/or Shtk domain proteins in nematode larvae have yet to be elucidated, tyrosinases of adult stages, (a type of oxidoreductase) assist in the cross-linking of proteins during eggshell production [86, 87]. Congruent with our gene ontology analysis of the *A*. *ceylanicum* L3 secretome and uniquely enriched therein, some tyrosinases participate in the melanin synthesis. *Teladorsagia circumcincta* appears to upregulate melanin production in response to direct sunshine and melanization may protect free-living larvae from environmental UV and genotoxicity [88]. More research is needed to determine the functional implications of tyrosinases and Shtk domain proteins, as one of the most abundant proteins in the adult *N*. *americanus* secretome was a Shtk protein without any tyrosinase annotation, and sequences associated with both functional terms featured prominently within a few different protein compartments in this study.

Transthyretin-like or transthyretin-related protein orthologs were detected from both development stages of *N*. *americanus* and *A*. *ceylanicum*, and the adult-specific secretome also included proteins belonging to this family (**Table 4B** and **4C**). Notably, transthyretins were not detected uniquely in the L3 secretome. In vertebrates, transthyretins function as a transporter of thyroid hormones but transthyretin-like proteins in nematodes, despite containing a similar transthyretin-like domain, do not appear to demonstrate thyroid binding properties [89, 90]. Transthyretin-like proteins and transthyretin-related proteins of nematodes have been implicated in biological processes such as larval development, protection against oxidative stress and the clearance of apoptotic cells [91–93]. *H*. *contortus* transthyretin also showed promise as a vaccine candidate, inducing a marked antigen-specific antibody response and reduction in parasite load in goats [94].

A FAR ortholog from *N*. *americanus* and *A*. *ceylanicum* was detected in both the L3 and adult stage ESPs (**Table 4C**), and a separate FAR (FAR-1) was identified among the more abundant adult-specific secretome proteins in both species (**Table 4B**). In addition, we identified highly abundant L3-specific FAR proteins that were unique to each species (**Table 1A** and **1B**). FARs bind lipids and retinol and are crucial for scavenging fatty acids and other lipid-based molecules from the host as nematode parasites have a limited capacity for lipid synthesis [95–97]. FARs have been described to be immunogenic [98,99] or immunomodulatory, and it was postulated that the latter property may be was facilitated by the ability of FAR to bind lipid-based signaling molecules, which interferes with their associated immune pathways [71,97].

Several of the orthologous secretome proteins identified in this study have minimal to no functional annotation assigned by any of the annotation tools. Given their interspecies overlap, these proteins could be implicated in crucial hookworm processes and further investigation into these proteins could yield novel insights into hookworm biology. We note, however, that due to the draft nature of these nematode genomes, some proteins and orthologous matches may have been missed from the proteomic identification altogether. Nonetheless, based on BUSCO [100] genome completeness analysis in earlier reports, we expect to have annotated 97.4% of all *N*. *americanus* proteins [15] and 94.5% of all *A*. *ceylanicum* proteins [16]. Hence, we do not expect a substantial number of missing annotations. It is also important to note that data generated by this study may be limited by the lack of biological replicates as it was difficult to culture a large number of L3 for ESP production due to the low number of L3 the participants were originally infected with and sample loss through processing. Further, only one participant out of six developed a patent *A*. *ceylanicum* infection that was sufficient to generate an adequate amount of L3 required for ESP production. A larger study in the future with more participants could overcome this limitation and further enrich this dataset through the production and analysis of a larger quantity of ESP. Moreover, biological replicates for adult hookworm ES products were also omitted due to the difficulty in obtaining sufficient number of adult worms in hamsters. The percentage of larvae that mature to adulthood in hamsters is low, and we had ethical concerns around the number of hamsters required to generate larger quantities of ES products.

Finally, comparisons to ESPs secreted from the cercarial stage of the flatworm *S*. *mansoni* provided a broader phylogenetic perspective of the data, since schistosomes also enter the host by invasion through the skin by the cercariae stage [39]. This analysis allowed for the phylogenetic categorization of several sets of proteins that appear to be very important for skin penetration, including helminth-conserved skin penetration ESPs (especially enolase and aldolase), nematode-specific proteins (transthyretin proteins, FAR-1, galectins, and others as described above) and enolases as very abundant trematode-specific proteins.

## 5 Conclusion

We identified and characterized the ES products from two developmental stages of *N*. *americanus* and *A*. *ceylanicum*. This appears to be the first report of the ES proteome from the L3 stage of any species of *Necator* or *Ancylostoma*. We compared the functional profiles from each respective life stage and describe several differences in terms of protein families and individual proteins. This revealed clear divergence in ESP profiles between life stages and between species, which is not unexpected given the variations in their behavior. As the proteins from the L3 have not been characterized from the two species, these differences in secretome proteins may represent distinct biological differences with implications for disease prevention and management. In addition, we identified three novel protein groups of interest from reciprocal hits between a combination of the species and life stages incorporating comparisons with previously published data sets. These protein groups identified likely represent perform essential common functions and processes in the hookworms due to their shared orthology. The similarities and differences highlighted here could both benefit and aid future research into a deeper understanding of these parasites and novel interventions to reduce their human impact.

### Disclaimer

The views, opinions and/or findings expressed are those of the authors and should not be interpreted as representing the official views or policies of the Department of Defense or the U.S. Government.

## Supporting information

S1 Table Functional annotations, proteomic detection, relative gene expression and BLAST results for the *N*. *americanus* genes/proteins. (XLSB)

S2 Table Functional annotations, proteomic detection, relative gene expression and BLAST results for the *A*. *ceylanicum* genes/proteins. (XLSB)

S3 Table Functional annotations, relative gene expression levels and proteomic detection levels for gene sets of interest. (**A**) 91 proteins detected only in L3 stage (not in adult) *N*. *americanus*, (**B**) 41 proteins detected in L3 stage (not in adult) *A*. *ceylanicum*, (**C**) 94 proteins detected only in adult stage (not in L3) *N*. *americanus*, (D) 496 proteins detected in adult stage (not in L3) *A*. *ceylanicum*, (**E**) 12 proteins detected in both L3 and adult stages of *N*. *americanus*, (**F**) 16 proteins detected in L3 and adult stages of *A*. *ceylanicum*, (**G**) 15 proteins detected only in L3 stage (not adult) of both *N*. *americanus* and *A*. *ceylanicum*, (**H**) five proteins detected in adult and L3 stages of both *N*. *americanus* and *A*. *ceylanicum* and (**I**) 16 proteins detected only in adult stage (not in L3) of both *N*. *americanus* and *A*. *ceylanicum*.
(XLSB)

S4 Table Significantly enriched InterPro domains, KEGG pathways and Gene Ontology Molecular Function and Biological Process terms for protein sets of interest. (**A**) The 91 proteins only detected in L3 of *N*. *americanus*, (**B**) the 41 proteins detected in L3 only of *A*. *ceylanicum*, (**C**) the 94 proteins only detected in adult *N*. *americanus* and (**D**) the 496 proteins detected only in adult *A*. *ceylanicum*.
(XLSX)
